# Performance of ChatGPT in Board Examinations for Specialists in the Japanese Ophthalmology Society

**DOI:** 10.7759/cureus.49903

**Published:** 2023-12-04

**Authors:** Daiki Sakai, Tadao Maeda, Atsuta Ozaki, Genki N Kanda, Yasuo Kurimoto, Masayo Takahashi

**Affiliations:** 1 Department of Ophthalmology, Kobe City Eye Hospital, Kobe, JPN; 2 Department of Ophthalmology, Kobe City Medical Center General Hospital, Kobe, JPN; 3 Department of Surgery, Division of Ophthalmology, Kobe University Graduate School of Medicine, Kobe, JPN; 4 Department of Ophthalmology, Mie University Graduate School of Medicine, Tsu, JPN; 5 Laboratory for Biologically Inspired Computing, RIKEN Center for Biosystems Dynamics Research, Kobe, JPN

**Keywords:** chatgpt, board examination, ophthalmology, large language models, generative artificial intelligence, artificial intelligence

## Abstract

We investigated the potential of ChatGPT in the ophthalmological field in the Japanese language using board examinations for specialists in the Japanese Ophthalmology Society. We tested GPT-3.5 and GPT-4-based ChatGPT on five sets of past board examination problems in July 2023. Japanese text was used as the prompt adopting two strategies: zero- and few-shot prompting. We compared the correct answer rate of ChatGPT with that of actual examinees, and the performance characteristics in 10 subspecialties were assessed. ChatGPT-3.5 and ChatGPT-4 correctly answered 112 (22.4%) and 229 (45.8%) out of 500 questions with simple zero-shot prompting, respectively, and ChatGPT-4 correctly answered 231 (46.2%) questions with few-shot prompting. The correct answer rates of ChatGPT-3.5 were approximately two to three times lower than those of the actual examinees for each examination set (p = 0.001). However, the correct answer rates for ChatGPT-4 were close to approximately 70% of those of the examinees. ChatGPT-4 had the highest correct answer rate (71.4% with zero-shot prompting and 61.9% with few-shot prompting) in “blepharoplasty, orbit, and ocular oncology,” and the lowest answer rate (30.0% with zero-shot prompting and 23.3% with few-shot prompting) in “pediatric ophthalmology.” We concluded that ChatGPT could be one of the advanced technologies for practical tools in Japanese ophthalmology.

## Introduction

Generative artificial intelligence (AI) is a branch of AI that focuses on creating new, original content, such as text, images, or audio, by learning from vast existing data. This cutting-edge technology has attracted considerable interest in various fields, including healthcare [1]. Notably, ChatGPT, the most well-known generative AI developed by OpenAI, has demonstrated impressive performance, nearly exceeding the passing line of the United States Medical Licensing Exam [2]. OpenAI consistently updates and refines ChatGPT. Additionally, by optimizing their inputs, users can participate in elevating the performance of ChatGPT, a technique termed prompt engineering. The simplest prompting strategy is zero-shot prompting, which consists of instruction and a task without giving any specific examples of how to perform that task [3]. The performance may improve with few-shot prompting, in which we provide a few examples to hint at the type of task [4]. Based on powerful performance and high expectations for further improvement, various use cases of ChatGPT supporting professional decision-making or interactions between medical professionals and patients have been proposed [5].

Previous reports have indicated that ChatGPT can correctly answer at least half of the questions regarding specialized knowledge in ophthalmology [6-8]. Surprisingly, ChatGPT, without any specialized training, applies to fields of expertise, such as ophthalmology. This fact motivates us to customize ChatGPT with domain-specific training with actual clinical data and to develop clinically applicable levels of ChatGPT technology-based tools in ophthalmology. In general, ChatGPT is known to perform best in English because it has been trained with a larger volume of English data than other languages. Therefore, the performance of ChatGPT may be lower if other languages are used, even when discussing the same topic. This concern should be verified before attempting the research and development of ChatGPT-based tools since operations in local languages are advantageous for clinical applications in non-English-speaking countries. This study aimed to investigate the potential viability of ChatGPT in the Japanese language as an advanced technology for practical ophthalmological tools using board examinations for specialists in the Japanese Ophthalmology Society.

## Materials and methods

ChatGPT

ChatGPT (OpenAI, L.L.C., CA, USA) is available on the OpenAI website. We used GPT-3.5-based ChatGPT (model: GPT-3.5-turbo-0613; hereinafter referred to as “ChatGPT-3.5”) and GPT-4-based ChatGPT (model: GPT-4-0613; hereinafter referred to as “ChatGPT-4”) for this study in July 2023.

Board examination for a specialist in Japanese Ophthalmology Society

We used five sets of past board examination problems for specialists in the Japanese Ophthalmology Society (30th to 34th, available online, https://www.nichigan.or.jp/senmon/purpose/examination.html). The board examination consists of two sections: text-based general questions and clinical vignettes with relevant images (100 and 50 questions per examination set, respectively), and average correct answer rates of actual examinees for past examinations are available for each section. The question formats were either multiple-choice questions with five options for a single answer or multiple-response questions with five options requiring two or three answers. We chose general questions, which widely cover all topics in ophthalmology, to assess background knowledge of ChatGPT. Thus, ChatGPT was required to answer 500 text-based questions.

Analysis

The Japanese text was used for all prompts in this study. First, we evaluated the ChatGPT-3.5 with simple zero-shot prompting (July 11 and 12, 2023). Specifically, the questions were input by 10 points with a brief introduction: “You are an ophthalmologist. Please answer the following 10 questions” for a new chat for every prompt. Then, we evaluated the ChatGPT-4 with the same zero-shot prompting (July 18 and 19, 2023). In addition, few-shot prompting involving two examples of questions and answers obtained from the 29th board examination problems were assigned to ChatGPT-4 (July 20, 2023). Some answers from ChatGPT-4 with few-shot prompting were missing, possibly due to character limits, and were complemented by asking separately in a new chat.

We compared the correct answer rates of ChatGPT-3.5, ChatGPT-4, and the actual examinees. Average marks disclosed by the Japanese Ophthalmology Society were referred to for the correct answer rates of the actual examinees. In addition, the characteristics of the performance in 10 subspecialties: anatomy and physiology; blepharoplasty, orbit, and ocular oncology; cataract and refractive error; glaucoma; neuro-ophthalmology; ocular surface; pediatric ophthalmology; retina; strabismus and amblyopia; uveitis; were assessed. Correct answers were prepared, and the questions were divided into 10 subspecialties based on discussions among three board-certified ophthalmologists (D.S., A.O., and T.M.). Between-group analyses were performed using the Friedman test, following the Bonferroni test as post-hoc analyses. All statistical analyses were performed using the SPSS software package (version 28; IBM Corp., Armonk, NY, USA). The statistical significance of all tests was set at p ≤ 0.05.

Ethical consideration

This study was conducted at the Kobe City Eye Hospital but did not require approval by the ethics committee because no individual patient data were involved.

## Results

ChatGPT-3.5 and ChatGPT-4 correctly answered 112 (22.4%) and 229 (45.8%) out of 500 questions, respectively, with simple zero-shot prompting. Using few-shot prompting, ChatGPT-4 correctly answered 231 (46.2%) questions. The examination results of the ChatGPT-3.5 and -4 and the examinees are listed in Table 1.

**Table 1:** The examination results of ChatGPT and actual examinees. NA, not available

	Number of questions	Multiple-response questions	Number of correct answers (correct answer rate)			Average marks of examinees
			ChatGPT-3.5	ChatGPT-4	ChatGPT-4 (few-shot)	
34^th^	100	37	23	49	54	70.9
33^rd^	100	55	22	41	39	61.6
32^nd^	100	46	20	48	47	65.7
31^st^	100	40	25	47	48	63.4
30^th^	100	37	22	44	43	67.1
Average value	—	43	22.4	45.8	46.2	65.7

ChatGPT-4 recorded better correct answer rates than ChatGPT-3.5. The correct answer rates of ChatGPT-3.5 were approximately two to three times lower than those of the actual examinees (p = 0.001). However, the correct answer rates for ChatGPT-4 were close to approximately 70% of those of the examinees.

**Table 2:** Comparison of the correct answer rates between ChatGPTs and actual examinees. *Significant at p < 0.05.

	Average value of the correct answer rates from five examinations	p value	Post hoc test		
			ChatGPT-4	ChatGPT-4 (few-shot)	Actual examinees
ChatGPT-3.5	22.4	0.004*	0.300	0.518	0.001*
ChatGPT-4	45.8			1.000	0.518
ChatGPT-4 (few-shot)	46.2				0.300
Actual examinees	65.7				

Among the 500 questions, 285 were multiple-choice (single answer), and the remaining 215 were multiple response (two or three answers). ChatGPT-3.5, ChatGPT-4 with zero-shot prompting, and ChatGPT-4 with few-shot prompting correctly answered 76 (26.7%), 141 (49.5%), and 146 (51.2%) of the 285 multiple-choice questions (single answer) and 36 (17.4%), 88 (40.9%), and 85 (39.5%) of the 215 multiple-response questions (two or three answers), respectively. ChatGPT-3.5 had 23 (4.6%) out of 500 questions with an incorrect amount of answers (for example, the question instructed the answerers to pick two answers, but ChatGPT picked only one). ChatGPT-4 had fewer questions with an incorrect amount of answers (nine questions), and further improvements were observed using few-shot prompting (only two questions). The data regarding the performance of ChatGPT in different subspecialties are summarized in Table 3.

**Table 3:** Performance of ChatGPT in 10 subspecialties

Subspecialty	Number of questions	ChatGPT-3.5		ChatGPT-4		ChatGPT-4 (few-shot)	
		Number of correct answers	Correct answer rate (%)	Number of correct answers	Correct answer rate (%)	Number of correct answers	Correct answer rate (%)
Anatomy and physiology	53 (10.6%)	13	24.5	23	43.4	28	52.8
Blepharoplasty, orbit, and ocular oncology	21 (4.2%)	5	23.8	15	71.4	13	61.9
Cataract and refractive error	43 (8.6%)	7	16.3	18	41.9	14	32.6
Glaucoma	47 (9.4%)	14	29.8	24	51.1	23	48.9
Neuro-ophthalmology	40 (8.0%)	9	22.5	19	47.5	18	45.0
Ocular surface	61 (12.2%)	12	19.7	32	52.5	34	55.7
Pediatric ophthalmology	30 (6.0%)	4	13.3	9	30.0	7	23.3
Retina	81 (16.2%)	17	21.0	35	43.2	35	43.2
Strabismus and amblyopia	30 (6.0%)	7	23.3	10	33.3	9	30.0
Uveitis	32 (6.4%)	7	21.9	12	37.5	16	50.0
Others	62 (12.4%)	17	27.4	32	51.6	34	54.8

For ChatGPT-3.5, the highest correct answer rate (28.8%) was related to “glaucoma,” whereas the lowest correct answer rate (12.5%) was related to “pediatric ophthalmology.” ChatGPT-4 had the highest correct answer rate (71.4% with zero-shot prompting and 61.9% with few-shot prompting) in “blepharoplasty, orbit, and ocular oncology,” and the lowest answer rate (30.0% with zero-shot and 23.3% with few-shot prompting) in “pediatric ophthalmology.” The questions labeled as “others” included topics regarding ethics and legal issues or multiple-true-false questions in which different fields were mixed. There were 14 calculation problems with correct answer rates of 14.3%, 28.6%, and 14.3% for ChatGPT-3.5, ChatGPT-4 with zero-shot prompting, and ChatGPT-4 with few-shot prompting, respectively. An example of a chat log with ChatGPT consisting of 10 sets of questions and answers is shown as supplementary material (Supplementary Figure 1).

## Discussion

We made ChatGPT answer board examination questions for specialists in the Japanese Ophthalmology Society to investigate its performance regarding specialized knowledge of ophthalmology in the Japanese language. Most ophthalmologists in Japan are certified by the Japanese ophthalmological society after taking the board examination. Only professionals who have finished a four-year ophthalmology residency are entitled to take the board examination, the passing rate of which has ranged from 66.9% to 90.6% in the past five years. The average marks of the examinees could be indicative of the real level of general ophthalmology knowledge in clinical practice in Japan. Although the correct answer rate for ChatGPT-3.5 (22.4%) was significantly lower than that for the actual examinees, ChatGPT-4 achieved improved performance with the correct answer rate of a shade below 50%, which was equivalent to previous reports that used English professional examinations in ophthalmology.

The subspecialty assessment showed various correct answer rates for ChatGPT-4, ranging 20%-70%. Moreover, ChatGPT-4 had the highest correct rate of over 60% for “blepharoplasty, orbit, and ocular oncology,” which was sufficient to independently reach the passing line. ChatGPT-3.5 and -4 had the worst performance for “pediatric ophthalmology.” The questions for “pediatric ophthalmology encompassed rare diseases, which could be potential targets for training to refine ChatGPT for professional applications. The correct answer rate for the calculation problems was low, which is considered a limitation of the current version of ChatGPT. Questions on “cataract and refractive error” and “strabismus and amblyopia” accounted for most of the calculation problems (seven and four of 14 questions, respectively), and this is likely related to the relatively low correct answer rates. ChatGPT performed better in answering multiple-choice questions with a single answer than in responding to multiple-response questions requiring two or three answers, which appears to align with the level of question difficulty. Interestingly, ChatGPT sometimes picked an incorrect amount of answers for the multiple-response questions. Prompt modification (few-shot prompting) was useful in reducing mistakes in answering the correct amount of answers, although it did not drastically change the correct answer rate in this study.

Mihalache et al. reported that ChatGPT-3.5 correctly answered 46% of the OphthoQuestions practice questions for US board certification examination preparation [6]. Antaki et al. tested the performance of ChatGPT-4 on preparation questions for a US board examination from OphthoQuestions and the Basic and Clinical Science Course Self-Assessment Program; the correct answer rates were 49.2% and 59.4%, respectively [7]. Raimondi et al. reported a comparable performance of ChatGPT-3.5 in the Fellowship of the Royal College of Ophthalmologists (FRCOphth) exams in the UK with a correct answer rate of 49.6%; moreover, they showed that ChatGPT-4 had better performance with a correct answer rate of 79.1% (88.4% with prompt modification) [8]. It is uncertain whether the difficulty levels of board examinations across different countries are comparable, but the performance of the latest ChatGPT (GPT-4-0613) in the Japanese language compares favorably with that in English. We confirmed that ChatGPT can use the Japanese language with background knowledge in ophthalmology, which suggests its promising potential as one of the advanced technologies for developing practical ophthalmological tools. In previous studies and our study, ChatGPT-4 consistently demonstrated higher performance than ChatGPT-3.5. ChatGPT-4, the most advanced system developed by OpenAI to date, is currently accessible only through a subscription to ChatGPT Plus at the price of $20 per month. An earlier model of ChatGPT-3.5 is available for free. ChatGPT-4 is based on the foundational architecture of ChatGPT-3.5 but is enhanced with advanced training using a broader, more recent, and diverse dataset. Given its enhanced performance in the field of ophthalmology, ChatGPT-4 is the preferred choice for further investigation.

ChatGPT passed the United States Medical Licensing Exam without specialized training, demonstrating its substantial knowledge in the general medical field [2,9]. This inspired us to apply this technology to medical education or clinical practice. In addition to the ophthalmology field, other specialized fields have been exploring ChatGPT’s capabilities and have seen promising prospects. ChatGPT-4 has demonstrated performance that closely approaches the passing line for the American board examination in orthopedics [10] and the German board examination in otolaryngology [11], with correct answer rates of 73.6% and 57%, respectively. Another report showed that ChatGPT could provide decent responses to commonly asked questions regarding plastic surgery (breast augmentation) [12]. As the specialized medical field becomes increasingly independent, we believe that domain-specific training, curated by experts in each area, is the next step in developing ChatGPT-based tools for clinical practice. Domain-specific training using actual clinical data must be required, in which we should carefully address ethical, legal, and practical considerations to prevent any errors in treatment or diagnosis [13,14]. AI has good compatibility with the field of ophthalmology, taking advantage of imaging-based diagnostics and high-volume medical examination data, supported by the highest number of outpatient visits among all medical specialties. The Japan Ocular Imaging Registry, created by the Japanese Ophthalmological Society, has been storing images and other data since 2017 [15], which could be a vital resource for training data. Meanwhile, subspecialties with less abundant examination data such as “pediatric ophthalmology” or “strabismus and amblyopia” were associated with relatively low correct answer rates in this study; therefore, these subspecialties could be important targets for improvement in ChatGPT’s application.

Contrary to the expected drawback of using the non-English language, the performances in the Japanese and English languages were comparable, particularly in the latest version of ChatGPT-4 (July 2023). The field of generative AI is dynamic and actively progressing; therefore, the performance of ChatGPT can improve even in the short term. We believe that keeping pace with this progress and re-examining it as appropriate is crucial. ChatGPT has several limitations that need to be monitored. First, the current ChatGPT cannot access specialized literature databases such as PubMed, and the inability to make references or citations is a major concern, leading to hesitation in its use in clinical practice. Consequently, ChatGPT was unable to process images during the study period. However, OpenAI recently launched an updated version of ChatGPT with image input capabilities. Future research should assess the reliance on image processing capabilities, which are strikingly important in the field of ophthalmology.

## Conclusions

ChatGPT could be one of the advanced technologies for practical tools in Japanese ophthalmology. Its performance on the Japanese ophthalmology board examination was satisfactory, and our results serve as a fundamental basis for considering its practical application using non-English language. The next study will explore its performance after domain-specific training with actual clinical data.

## Figures and Tables

**Figure 1:**
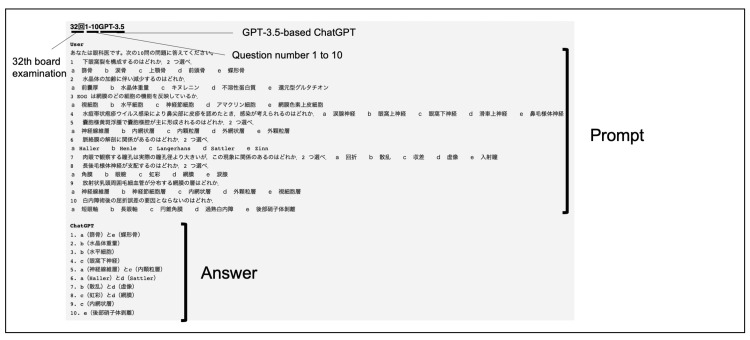
The chat log with ChatGPT. An example of a chat log consisting of 10 sets of questions and answers.
